# 4-(2,3-Di­chloro­phen­yl)piperazin-1-ium picrate

**DOI:** 10.1107/S2414314621003795

**Published:** 2021-04-13

**Authors:** Udhayasuriyan Sathya, Jeyaraman Selvaraj Nirmal Ram, Sundaramoorthy Gomathi, Shyamaladevi Ramu, Samson Jegan Jennifer, Abdul Razak Ibrahim

**Affiliations:** a Centre for Research and Development, PRIST Deemed to be University, Thanjavur, 613 403, Tamil Nadu, India; b Department of Chemistry, Periyar Maniammai Institute of Science and Technology, Thanjavur 613 403, Tamil Nadu, India; c X-ray Crystallography Unit, School of Physics, University Sains Malaysia, 11800, USM, Penang, Malaysia; d X-ray Crystallography Unit, School of Physics, Universiti Sains Malaysia, 11800, USM, Penang, Malaysia; Howard University, USA

**Keywords:** 1-(2,3-di­chloro­phen­yl)piperazinium picrate, crystal structure, supra­molecular inter­action

## Abstract

The title compound, C_6_H_2_N_3_O_7_^−^·C_10_H_13_Cl_2_N_2_^+^, crystallizes with one 1-(2,3-di­chloro-phen­yl)piperazine (DP) cation and one picrate (PA) anion in the asymmetric unit. In the crystal structure, the DP cation and PA anion are inter­connected *via* several N—H⋯O and C—H⋯O hydrogen bonds. The DP cation and PA anion are further connected through C—Cl⋯π [3.8201 (4), 3.7785 (4) Å] and N—O⋯π [3.7814 (4) Å] inter­actions. The DP cations are further inter­connected *via* a weak inter­molecular Cl⋯Cl [3.2613 (4) Å] halogen–halogen inter­action. The combination of these supra­molecular inter­actions leads to a herringbone like supra­molecular architecture.

## Abstract 2

The title compound, C_6_H_2_N_3_O_7_^−^·C_10_H_13_Cl_2_N_2_^+^, crystallizes with one 1-(2,3-di­chloro­phen­yl)piperazine cation and one picrate anion in the asymmetric unit. In the crystal structure, the cations and anions are inter­connected *via* several N—H⋯O and C—H⋯O hydrogen bonds.

## Graphical abstract

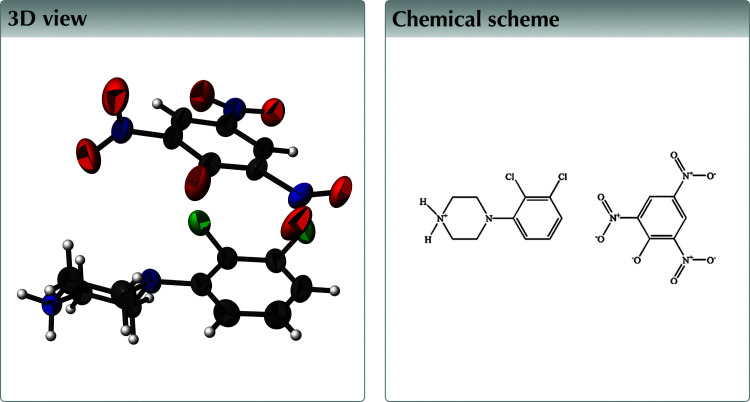

## Structure description

1-(2,3-Di­chloro­phen­yl)piperazine (DP), a precursor in the synthesis of potent drugs such as aripiperazole (AP) (Oshiro *et al.*, 1998 ▸), is used as an anti­psychotic drug for the treatment of schizophrenia (Braun *et al.*, 2009 ▸; Frank *et al.*, 2007 ▸). A survey of the Cambridge Structural Database (CSD version 5.40, updates of May 2019; Groom *et al.*, 2016 ▸) shows that there are no reports of salt and co-crystal forms of this compound. We herein report the crystal structure of a new solid form of DP, 1-(2,3-di­chloro-phen­yl)-piperazinium picrate (**1**).

**Figure 1:**
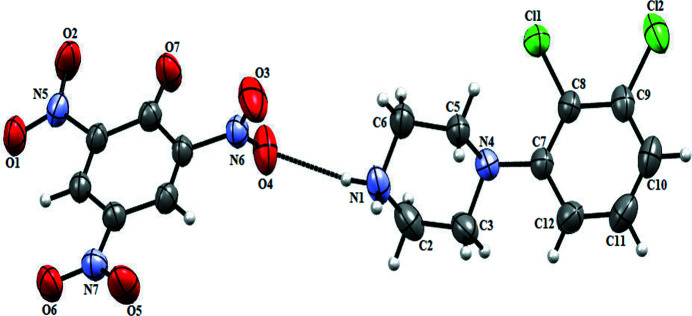
The title compound shown with 50% probability ellipsoids. The hydrogen bond is shown as a dashed line.

The title salt, **1**, crystallizes in the monoclinic *P*2_1_/*n* space group. The asymmetric unit contains one (DP) cation and one picrate (PA) anion as shown in Fig. 1 ▸. In **1**, the pyrazine ring of the cation mol­ecule adopts a chair conformation with N—H and C—H bonds in axial–axial and equatorial–equatorial positions (Singh *et al.*, 2015 ▸; Maia *et al.*, 2012 ▸).

**Figure 2:**
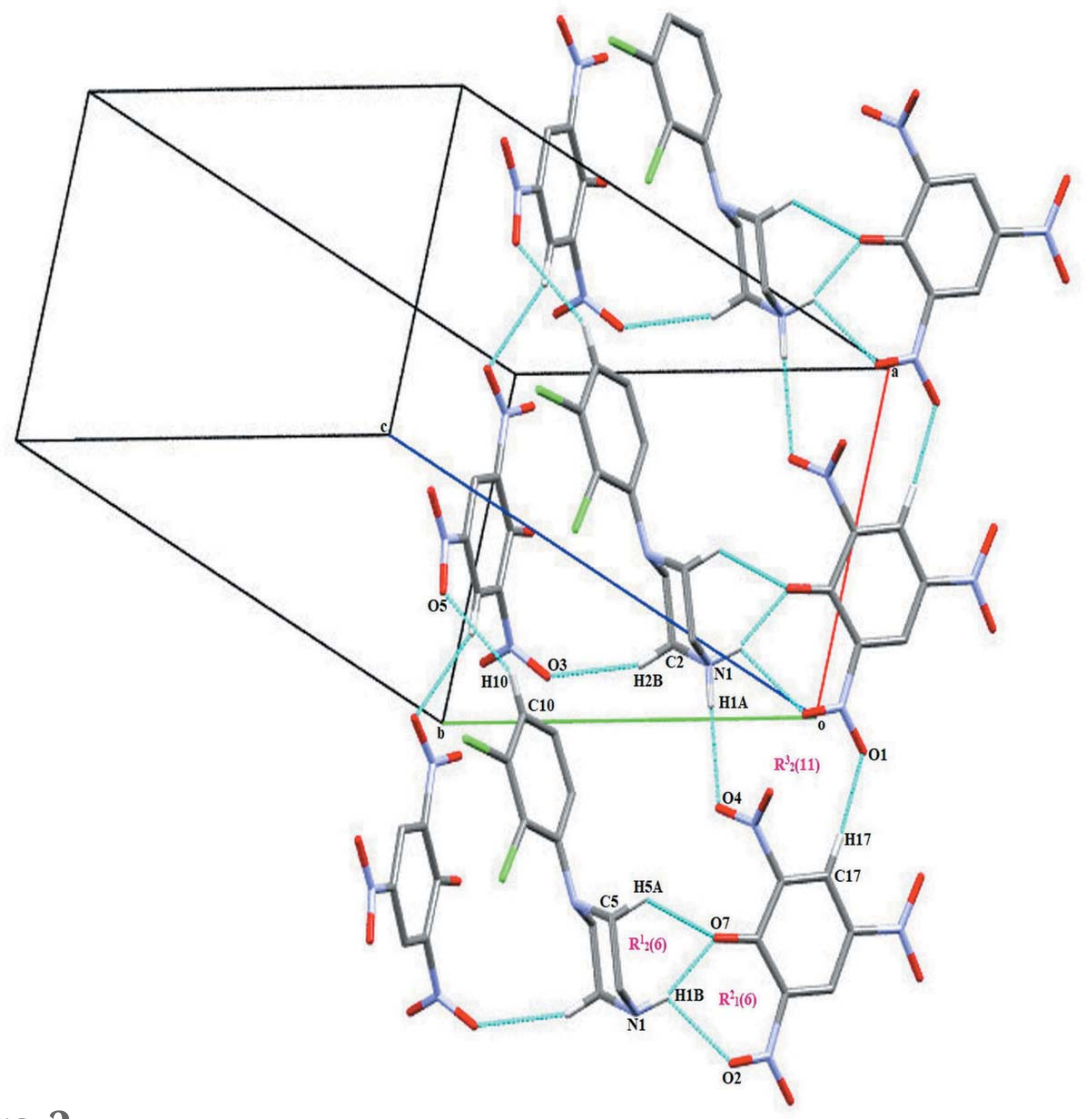
A view of the N—H⋯O and C—H⋯O hydrogen-bonded packing pattern of the title salt.

**Figure 3:**
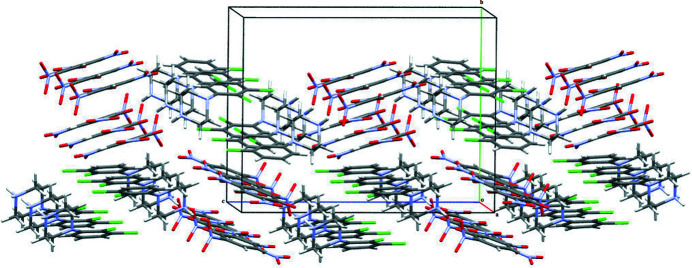
The three dimensional herring bone supra­molecular architecture viewed along the *a* and *c* axis.

**Figure 4:**
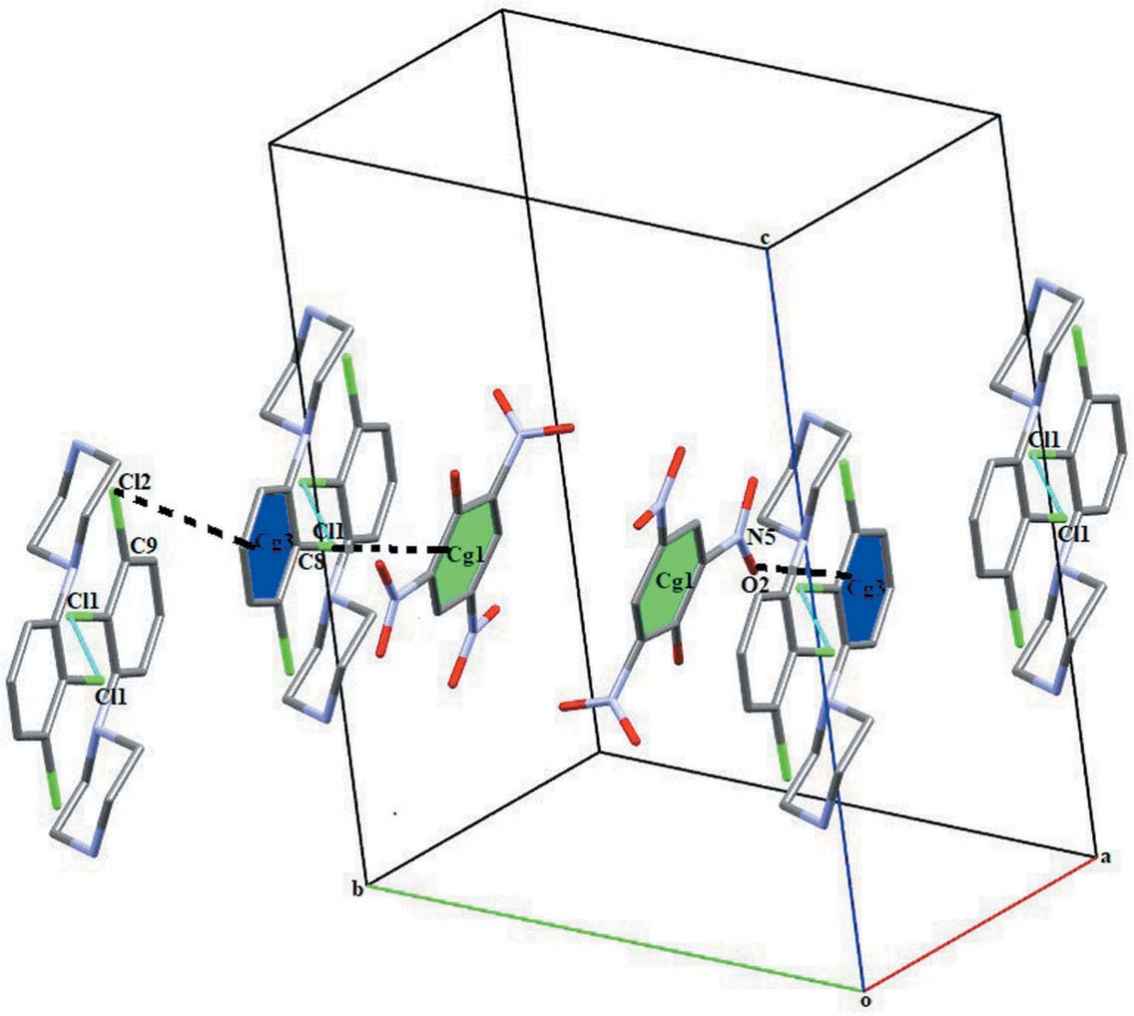
A view of the C—Cl⋯ π and N—O⋯π inter­actions involving the phenyl rings of the cation and anion (at symmetry positions *x*, *y*, *z* and 1 − *x*, −*y*, 1 − *z*) and the weak inter­molecular Cl⋯Cl halogen–halogen bond.

The protonated DP cation inter­acts with the neighbouring deprotonated PA anions *via* N1—H1*A*⋯O4^i^, N1—H1*B*⋯O2^ii^ and N1—H1*B*⋯O7^ii^ hydrogen bonds and C2—H2*B*⋯O3, C5—H5*A*⋯O7^ii^, C10—H10⋯O5^iii^ and C17—H17⋯O1^iv^ hydrogen bonds (Table 1 ▸). The crystal packing is shown in Fig. 2 ▸. Each DP cation is surrounded by four PA anions. The combination of N1—H1*B*⋯O7, N1—H1*B*⋯O2 and C5—H5*A*⋯O7 inter­actions between the ions leads to the formation of six-membered rings with graph-set notation 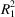 (6) and 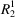 (6) (Bernstein *et al.*, 1995 ▸; Motherwell *et al.*, 2000 ▸). Atom H1*B* of the amino group (N1) acts as a bifurcated donor to the O atoms of the deprotonated O1 carbonyl and O2 nitro groups of the PA anion. Inversion-related cation–anion pairs are also linked through N1—H1*A*⋯O4, N1—H1*B*⋯O2 and C17—H17⋯O1 hydrogen bonds, forming an 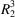 (11) ring motif. Adjacent DP cations and PA anions are further connected through C8—Cl1⋯π (phenyl ring of PA anion), C9—H9⋯ π (phenyl ring of DP cation) and N5—O2⋯π (phenyl ring of DP cation) inter­actions [C—Cl⋯*Cg*1, C—Cl⋯*Cg*3^v^ and N—O⋯*Cg*3; symmetry codes: (v) 1 − *x*, 2 − *y*, 1 − *z*] with C⋯π distances of 3.8201 (4) and 3.7785 (4) Å, and N⋯π = 3.782 (2) Å, with C—Cl⋯π angles of 74.15 (7) and 76.91 (7)° and an N—O⋯π angle of 68.80 (12)°. The combination of N—H⋯O and C—H⋯O hydrogen bonds and C—Cl⋯π and N—O⋯π inter­actions leads to the formation of a three-dimensional supra­molecular herringbone architecture, which propagates along the *a-* and *c*-axis directions (Fig. 3 ▸). Additionally, the DP cations are also connected through weak inter­molecular halogen–halogen Cl1⋯Cl1(7 − *x*, 2 − *y*, -*z)* inter­actions [3.2613 (4) Å] (Fig. 4 ▸).

## Synthesis and crystallization

**Figure 5:**
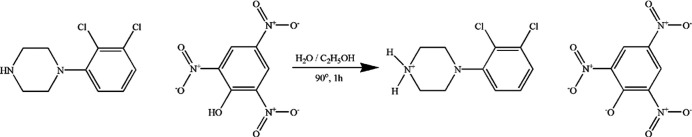
Reaction scheme.

1-(2,3-Di­chloro­phen­yl)piperazine (DP) (0.0577 mg, 0.25 mmol) and picric acid (PA) (0.05727 mg, 0.25 mmol) were dissolved independently in water and ethanol. The reactants were then mixed together in a 100 ml beaker and heated over a water bath at 90°C for 1 h (Fig. 5 ▸). The clear reaction mixture was then left aside for crystallization at room temperature. After a few days, yellow-coloured plate-like crystals formed were separated out form the mother solution.

## Refinement

Crystal data, data collection and structure refinement details are summarized in Table 2 ▸.

## Supplementary Material

Crystal structure: contains datablock(s) I. DOI: 10.1107/S2414314621003795/bv4037sup1.cif

Structure factors: contains datablock(s) I. DOI: 10.1107/S2414314621003795/bv4037Isup2.hkl

Supporting information file. DOI: 10.1107/S2414314621003795/bv4037Isup3.cml

CCDC reference: 2076126

Additional supporting information:  crystallographic information; 3D view; checkCIF report

## Figures and Tables

**Table 1:** Hydrogen-bond geometry (Å, °)

*D*—H⋯*A*	*D*—H	H⋯*A*	*D*⋯*A*	*D*—H⋯*A*
N1—H1*A*⋯O4^i^	0.89	2.25	3.134 (2)	171
N1—H1*B*⋯O2^ii^	0.89	2.28	2.828 (3)	119
N1—H1*B*⋯O7^ii^	0.89	1.84	2.695 (2)	159
C2—H2*B*⋯O3	0.97	2.59	3.444 (3)	148
C5—H5*A*⋯O7^ii^	0.97	2.59	3.287 (2)	129
C10—H10⋯O5^iii^	0.93	2.56	3.399 (3)	151
C17—H17⋯O1^iv^	0.93	2.50	3.348 (2)	152

Symmetry codes: (i) 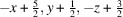 ; (ii) 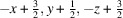 ; (iii) 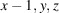 ; (iv) 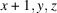 .

**Table 2:** Experimental details

Crystal data	
Chemical formula	C_10_H_13_Cl_2_N_2_^+^·C_6_H_2_N_3_O_7_^−^
*M*_r_	460.23
Crystal system, space group	Monoclinic, *P*2_1_/*n*
Temperature (K)	293
*a*, *b*, *c* (Å)	7.9855 (9), 13.5742 (15), 17.6103 (19)
β (°)	91.463 (4)
*V* (Å^3^)	1908.3 (4)
*Z*	4
Radiation type	Mo *K*α
μ (mm^−1^)	0.39
Crystal size (mm)	0.40 × 0.35 × 0.20

Data collection	
Diffractometer	Bruker APEXII CCD
Absorption correction	Multi-scan (*SADABS*; Bruker, 2009 ▸)
No. of measured, independent and observed [*I* > 2σ(*I*)] reflections	72319, 5581, 3798
*R*_int_	0.060
(sin θ/λ)_max_ (Å^−1^)	0.704

Refinement	
*R*[*F* ^2^ > 2σ(*F* ^2^)], *wR*(*F* ^2^), *S*	0.052, 0.150, 1.01
No. of reflections	5581
No. of parameters	271
H-atom treatment	H-atom parameters constrained
Δρ_max_, Δρ_min_ (e Å^−3^)	0.41, −0.31

Computer programs: *APEX2* and *SAINT* (Bruker, 2009 ▸), *SHELXS97* (Sheldrick 2008 ▸), *SHELXL2018/3* (Sheldrick, 2015 ▸), *PLATON* (Spek, 2020 ▸), *Mercury* (Macrae *et al.*, 2020 ▸), POVRay (Cason, 2004 ▸) and *publCIF* (Westrip,2010 ▸).

## full crystallographic data

### Crystal data

**Table:**

C_10_H_13_Cl_2_N_2_^+^·C_6_H_2_N_3_O_7_^−^	*F*(000) = 944
*M**_r_* = 460.23	*D*_x_ = 1.602 Mg m^−^^3^
Monoclinic, *P*2_1_/*n*	Mo *K*α radiation, λ = 0.71073 Å
*a* = 7.9855 (9) Å	Cell parameters from 5581 reflections
*b* = 13.5742 (15) Å	θ = 1.9–30.0°
*c* = 17.6103 (19) Å	µ = 0.39 mm^−^^1^
β = 91.463 (4)°	*T* = 293 K
*V* = 1908.3 (4) Å^3^	Plate, yellow
*Z* = 4	0.40 × 0.35 × 0.20 mm

### Data collection

**Table:**

Bruker APEXII CCD diffractometer	3798 reflections with *I* > 2σ(*I*)
φ and ω scans	*R*_int_ = 0.060
Absorption correction: multi-scan (SADABS; Bruker, 2009)	θ_max_ = 30.0°, θ_min_ = 1.9°
	*h* = −11→11
72319 measured reflections	*k* = −19→19
5581 independent reflections	*l* = −24→24

### Refinement

**Table:**

Refinement on *F*^2^	Primary atom site location: structure-invariant direct methods
Least-squares matrix: full	Secondary atom site location: difference Fourier map
*R*[*F*^2^ > 2σ(*F*^2^)] = 0.052	Hydrogen site location: inferred from neighbouring sites
*wR*(*F*^2^) = 0.150	H-atom parameters constrained
*S* = 1.01	*w* = 1/[σ^2^(*F*_o_^2^) + (0.0739*P*)^2^ + 0.6029*P*] where *P* = (*F*_o_^2^ + 2*F*_c_^2^)/3
5581 reflections	(Δ/σ)_max_ < 0.001
271 parameters	Δρ_max_ = 0.41 e Å^−^^3^
0 restraints	Δρ_min_ = −0.31 e Å^−^^3^

### Special details

**Table:**

Geometry. All esds (except the esd in the dihedral angle between two l.s. planes) are estimated using the full covariance matrix. The cell esds are taken into account individually in the estimation of esds in distances, angles and torsion angles; correlations between esds in cell parameters are only used when they are defined by crystal symmetry. An approximate (isotropic) treatment of cell esds is used for estimating esds involving l.s. planes.

### Fractional atomic coordinates and isotropic or equivalent isotropic displacement parameters (Å^2^)

**Table:**

	*x*	*y*	*z*	*U*_iso_*/*U*_eq_	
Cl1	0.86754 (7)	0.90877 (4)	0.49364 (3)	0.05570 (16)	
Cl2	0.55596 (9)	0.87130 (5)	0.38716 (3)	0.06593 (19)	
O1	0.42341 (18)	0.61036 (13)	0.47240 (9)	0.0596 (4)	
O3	1.1299 (3)	0.66281 (15)	0.68775 (11)	0.0815 (6)	
O4	1.1043 (2)	0.50943 (13)	0.66777 (11)	0.0737 (5)	
O6	0.8856 (2)	0.70824 (13)	0.33400 (8)	0.0620 (4)	
O7	0.73482 (19)	0.56905 (14)	0.66344 (8)	0.0607 (4)	
N1	1.0258 (2)	0.97533 (15)	0.77447 (10)	0.0557 (5)	
H1A	1.129102	0.979276	0.794458	0.067*	
H1B	0.958093	1.009367	0.804070	0.067*	
N4	0.8056 (2)	0.90163 (12)	0.65808 (9)	0.0448 (4)	
N5	0.5018 (2)	0.60547 (13)	0.53316 (10)	0.0464 (4)	
N6	1.0712 (2)	0.59397 (13)	0.65270 (9)	0.0449 (4)	
N7	0.9808 (2)	0.70084 (14)	0.38929 (9)	0.0491 (4)	
C2	0.9720 (3)	0.8704 (2)	0.77267 (13)	0.0631 (6)	
H2A	0.968767	0.844980	0.824094	0.076*	
H2B	1.052353	0.831680	0.744979	0.076*	
C3	0.8019 (3)	0.86087 (18)	0.73514 (12)	0.0565 (5)	
H3A	0.769483	0.792039	0.732931	0.068*	
H3B	0.719974	0.896037	0.764531	0.068*	
C5	0.8499 (3)	1.00694 (15)	0.66127 (11)	0.0462 (4)	
H5A	0.768611	1.042218	0.690919	0.055*	
H5B	0.847524	1.034217	0.610346	0.055*	
C6	1.0229 (3)	1.01986 (18)	0.69695 (12)	0.0534 (5)	
H6A	1.105461	0.987896	0.665858	0.064*	
H6B	1.050319	1.089372	0.700291	0.064*	
C7	0.6619 (3)	0.88178 (14)	0.61170 (11)	0.0428 (4)	
C8	0.6754 (2)	0.88572 (14)	0.53246 (11)	0.0421 (4)	
C9	0.5373 (3)	0.86809 (15)	0.48480 (12)	0.0482 (5)	
C10	0.3843 (3)	0.84451 (17)	0.51519 (15)	0.0582 (6)	
H10	0.291238	0.833430	0.483512	0.070*	
C11	0.3714 (3)	0.83768 (19)	0.59227 (16)	0.0627 (6)	
H11	0.269194	0.820331	0.612601	0.075*	
C12	0.5069 (3)	0.85596 (18)	0.64071 (14)	0.0574 (6)	
H12	0.494641	0.851030	0.692952	0.069*	
C13	0.7830 (2)	0.59718 (14)	0.59988 (11)	0.0408 (4)	
C14	0.6823 (2)	0.61733 (14)	0.53291 (11)	0.0393 (4)	
C15	0.7479 (2)	0.64827 (14)	0.46521 (11)	0.0399 (4)	
H15	0.677360	0.659033	0.423206	0.048*	
C16	0.9173 (2)	0.66320 (14)	0.45975 (10)	0.0406 (4)	
C17	1.0258 (2)	0.64607 (15)	0.52161 (11)	0.0425 (4)	
H17	1.140531	0.656262	0.518004	0.051*	
C18	0.9590 (2)	0.61409 (14)	0.58717 (11)	0.0398 (4)	
O2	0.43209 (19)	0.59195 (15)	0.59333 (10)	0.0695 (5)	
O5	1.1286 (2)	0.72559 (16)	0.38803 (9)	0.0718 (5)	

### Atomic displacement parameters (Å^2^)

**Table:**

	*U*^11^	*U*^22^	*U*^33^	*U*^12^	*U*^13^	*U*^23^
Cl1	0.0499 (3)	0.0718 (4)	0.0456 (3)	−0.0124 (2)	0.0050 (2)	−0.0060 (2)
Cl2	0.0769 (4)	0.0705 (4)	0.0493 (3)	−0.0001 (3)	−0.0201 (3)	−0.0011 (3)
O1	0.0332 (7)	0.0855 (12)	0.0594 (9)	−0.0006 (7)	−0.0084 (7)	0.0115 (8)
O3	0.0942 (14)	0.0746 (12)	0.0738 (12)	−0.0009 (10)	−0.0364 (11)	−0.0154 (10)
O4	0.0748 (12)	0.0611 (11)	0.0835 (12)	0.0018 (9)	−0.0331 (10)	0.0162 (9)
O6	0.0556 (9)	0.0880 (12)	0.0424 (8)	0.0026 (8)	−0.0011 (7)	0.0138 (8)
O7	0.0418 (8)	0.0957 (12)	0.0445 (8)	−0.0086 (8)	0.0000 (6)	0.0179 (8)
N1	0.0409 (9)	0.0817 (13)	0.0441 (9)	0.0083 (9)	−0.0063 (7)	−0.0172 (9)
N4	0.0456 (9)	0.0503 (9)	0.0384 (8)	−0.0041 (7)	−0.0035 (7)	−0.0005 (7)
N5	0.0334 (8)	0.0518 (10)	0.0538 (10)	0.0049 (7)	0.0004 (7)	0.0101 (7)
N6	0.0357 (8)	0.0566 (11)	0.0423 (8)	−0.0009 (7)	−0.0037 (6)	0.0030 (7)
N7	0.0441 (9)	0.0610 (11)	0.0424 (9)	−0.0009 (8)	0.0037 (7)	0.0038 (7)
C2	0.0720 (16)	0.0737 (16)	0.0431 (11)	0.0137 (12)	−0.0103 (10)	−0.0005 (10)
C3	0.0684 (14)	0.0595 (13)	0.0415 (10)	−0.0043 (11)	−0.0035 (10)	0.0036 (9)
C5	0.0433 (10)	0.0526 (11)	0.0424 (10)	−0.0050 (8)	−0.0037 (8)	−0.0040 (8)
C6	0.0433 (11)	0.0678 (14)	0.0492 (11)	−0.0053 (10)	−0.0006 (9)	−0.0087 (10)
C7	0.0431 (10)	0.0421 (10)	0.0432 (10)	−0.0052 (8)	−0.0017 (8)	−0.0038 (8)
C8	0.0396 (10)	0.0408 (10)	0.0458 (10)	−0.0052 (7)	−0.0017 (8)	−0.0028 (8)
C9	0.0514 (12)	0.0402 (10)	0.0523 (11)	0.0003 (8)	−0.0124 (9)	−0.0028 (8)
C10	0.0422 (11)	0.0549 (13)	0.0769 (15)	−0.0048 (9)	−0.0135 (10)	−0.0065 (11)
C11	0.0454 (12)	0.0611 (14)	0.0819 (17)	−0.0134 (10)	0.0058 (11)	−0.0061 (12)
C12	0.0545 (13)	0.0630 (14)	0.0551 (12)	−0.0126 (10)	0.0082 (10)	−0.0050 (10)
C13	0.0337 (9)	0.0480 (10)	0.0408 (9)	−0.0015 (7)	0.0012 (7)	0.0036 (8)
C14	0.0278 (8)	0.0473 (10)	0.0428 (9)	0.0024 (7)	−0.0005 (7)	0.0038 (8)
C15	0.0348 (9)	0.0449 (10)	0.0397 (9)	0.0026 (7)	−0.0044 (7)	0.0028 (7)
C16	0.0356 (9)	0.0483 (10)	0.0380 (9)	−0.0005 (7)	0.0019 (7)	0.0035 (7)
C17	0.0300 (9)	0.0522 (11)	0.0453 (10)	−0.0007 (7)	0.0002 (7)	0.0016 (8)
C18	0.0311 (9)	0.0497 (10)	0.0383 (9)	0.0009 (7)	−0.0052 (7)	0.0021 (7)
O2	0.0348 (7)	0.1106 (15)	0.0634 (10)	0.0098 (8)	0.0102 (7)	0.0282 (9)
O5	0.0486 (9)	0.1141 (15)	0.0533 (9)	−0.0199 (9)	0.0101 (7)	0.0089 (9)

### Geometric parameters (Å, º)

**Table:**

Cl1—C8	1.724 (2)	C3—H3B	0.9700
Cl2—C9	1.730 (2)	C5—C6	1.513 (3)
O1—N5	1.227 (2)	C5—H5A	0.9700
O3—N6	1.208 (2)	C5—H5B	0.9700
O4—N6	1.206 (2)	C6—H6A	0.9700
O6—N7	1.224 (2)	C6—H6B	0.9700
O7—C13	1.252 (2)	C7—C12	1.396 (3)
N1—C2	1.488 (3)	C7—C8	1.403 (3)
N1—C6	1.493 (3)	C8—C9	1.389 (3)
N1—H1A	0.8900	C9—C10	1.384 (3)
N1—H1B	0.8900	C10—C11	1.367 (4)
N4—C7	1.417 (3)	C10—H10	0.9300
N4—C3	1.467 (3)	C11—C12	1.383 (3)
N4—C5	1.474 (3)	C11—H11	0.9300
N5—O2	1.223 (2)	C12—H12	0.9300
N5—C14	1.451 (2)	C13—C14	1.436 (3)
N6—C18	1.468 (2)	C13—C18	1.447 (3)
N7—O5	1.228 (2)	C14—C15	1.380 (3)
N7—C16	1.445 (2)	C15—C16	1.373 (3)
C2—C3	1.501 (3)	C15—H15	0.9300
C2—H2A	0.9700	C16—C17	1.394 (3)
C2—H2B	0.9700	C17—C18	1.356 (3)
C3—H3A	0.9700	C17—H17	0.9300
			
C2—N1—C6	111.74 (17)	N1—C6—H6B	109.9
C2—N1—H1A	109.3	C5—C6—H6B	109.9
C6—N1—H1A	109.3	H6A—C6—H6B	108.3
C2—N1—H1B	109.3	C12—C7—C8	117.72 (19)
C6—N1—H1B	109.3	C12—C7—N4	123.32 (19)
H1A—N1—H1B	107.9	C8—C7—N4	118.95 (17)
C7—N4—C3	115.25 (17)	C9—C8—C7	120.93 (19)
C7—N4—C5	113.38 (16)	C9—C8—Cl1	119.49 (16)
C3—N4—C5	109.94 (16)	C7—C8—Cl1	119.54 (15)
O2—N5—O1	122.01 (17)	C10—C9—C8	120.1 (2)
O2—N5—C14	119.57 (17)	C10—C9—Cl2	119.29 (17)
O1—N5—C14	118.42 (17)	C8—C9—Cl2	120.59 (17)
O4—N6—O3	122.95 (19)	C11—C10—C9	119.3 (2)
O4—N6—C18	118.42 (17)	C11—C10—H10	120.4
O3—N6—C18	118.60 (18)	C9—C10—H10	120.4
O6—N7—O5	122.73 (17)	C10—C11—C12	121.5 (2)
O6—N7—C16	119.20 (17)	C10—C11—H11	119.2
O5—N7—C16	118.06 (17)	C12—C11—H11	119.2
N1—C2—C3	110.43 (19)	C11—C12—C7	120.4 (2)
N1—C2—H2A	109.6	C11—C12—H12	119.8
C3—C2—H2A	109.6	C7—C12—H12	119.8
N1—C2—H2B	109.6	O7—C13—C14	127.87 (17)
C3—C2—H2B	109.6	O7—C13—C18	120.54 (17)
H2A—C2—H2B	108.1	C14—C13—C18	111.60 (16)
N4—C3—C2	109.6 (2)	C15—C14—C13	123.41 (16)
N4—C3—H3A	109.7	C15—C14—N5	115.83 (16)
C2—C3—H3A	109.7	C13—C14—N5	120.76 (16)
N4—C3—H3B	109.7	C16—C15—C14	120.11 (17)
C2—C3—H3B	109.7	C16—C15—H15	119.9
H3A—C3—H3B	108.2	C14—C15—H15	119.9
N4—C5—C6	110.14 (17)	C15—C16—C17	120.96 (16)
N4—C5—H5A	109.6	C15—C16—N7	118.66 (17)
C6—C5—H5A	109.6	C17—C16—N7	120.34 (17)
N4—C5—H5B	109.6	C18—C17—C16	117.94 (17)
C6—C5—H5B	109.6	C18—C17—H17	121.0
H5A—C5—H5B	108.1	C16—C17—H17	121.0
N1—C6—C5	109.00 (18)	C17—C18—C13	125.98 (17)
N1—C6—H6A	109.9	C17—C18—N6	118.89 (16)
C5—C6—H6A	109.9	C13—C18—N6	115.13 (16)
			
C6—N1—C2—C3	−55.7 (2)	C18—C13—C14—C15	0.4 (3)
C7—N4—C3—C2	169.33 (18)	O7—C13—C14—N5	−0.3 (3)
C5—N4—C3—C2	−61.0 (2)	C18—C13—C14—N5	−179.80 (17)
N1—C2—C3—N4	57.7 (2)	O2—N5—C14—C15	−169.69 (19)
C7—N4—C5—C6	−167.66 (17)	O1—N5—C14—C15	9.6 (3)
C3—N4—C5—C6	61.7 (2)	O2—N5—C14—C13	10.5 (3)
C2—N1—C6—C5	55.3 (2)	O1—N5—C14—C13	−170.20 (18)
N4—C5—C6—N1	−57.9 (2)	C13—C14—C15—C16	−1.3 (3)
C3—N4—C7—C12	20.8 (3)	N5—C14—C15—C16	178.92 (18)
C5—N4—C7—C12	−107.2 (2)	C14—C15—C16—C17	1.0 (3)
C3—N4—C7—C8	−157.64 (19)	C14—C15—C16—N7	−176.60 (18)
C5—N4—C7—C8	74.4 (2)	O6—N7—C16—C15	−7.9 (3)
C12—C7—C8—C9	2.4 (3)	O5—N7—C16—C15	171.0 (2)
N4—C7—C8—C9	−179.13 (18)	O6—N7—C16—C17	174.48 (19)
C12—C7—C8—Cl1	−175.49 (17)	O5—N7—C16—C17	−6.6 (3)
N4—C7—C8—Cl1	3.0 (3)	C15—C16—C17—C18	0.1 (3)
C7—C8—C9—C10	−1.2 (3)	N7—C16—C17—C18	177.69 (19)
Cl1—C8—C9—C10	176.69 (17)	C16—C17—C18—C13	−1.1 (3)
C7—C8—C9—Cl2	−179.03 (15)	C16—C17—C18—N6	178.99 (17)
Cl1—C8—C9—Cl2	−1.2 (2)	O7—C13—C18—C17	−178.8 (2)
C8—C9—C10—C11	−0.8 (3)	C14—C13—C18—C17	0.8 (3)
Cl2—C9—C10—C11	177.11 (18)	O7—C13—C18—N6	1.2 (3)
C9—C10—C11—C12	1.5 (4)	C14—C13—C18—N6	−179.27 (16)
C10—C11—C12—C7	−0.2 (4)	O4—N6—C18—C17	−103.0 (2)
C8—C7—C12—C11	−1.7 (3)	O3—N6—C18—C17	74.9 (3)
N4—C7—C12—C11	179.9 (2)	O4—N6—C18—C13	77.0 (2)
O7—C13—C14—C15	180.0 (2)	O3—N6—C18—C13	−105.0 (2)

### Hydrogen-bond geometry (Å, º)

**Table:**

*D*—H···*A*	*D*—H	H···*A*	*D*···*A*	*D*—H···*A*
N1—H1*A*···O4^i^	0.89	2.25	3.134 (2)	171
N1—H1*B*···O2^ii^	0.89	2.28	2.828 (3)	119
N1—H1*B*···O7^ii^	0.89	1.84	2.695 (2)	159
C2—H2*B*···O3	0.97	2.59	3.444 (3)	148
C5—H5*A*···O7^ii^	0.97	2.59	3.287 (2)	129
C10—H10···O5^iii^	0.93	2.56	3.399 (3)	151
C17—H17···O1^iv^	0.93	2.50	3.348 (2)	152

Symmetry codes: (i) −*x*+5/2, *y*+1/2, −*z*+3/2; (ii) −*x*+3/2, *y*+1/2, −*z*+3/2; (iii) *x*−1, *y*, *z*; (iv) *x*+1, *y*, *z*.
